# Is the surge in cesarean section rates during the COVID-19 pandemic truly substantiated?

**DOI:** 10.1186/s12884-024-06492-1

**Published:** 2024-04-12

**Authors:** Bakhtiar Piroozi, Ghobad Moradi, Kimya Khoramipoor, Hassan Mahmoodi, Farnaz Zandvakili, Ali Ebrazeh, Azad Shokri, Farhad Moradpour

**Affiliations:** 1 Social Determinants of Health Research Center, Research Institute for Health Development, Kurdistan University of Medical Sciences, Sanandaj, Iran; 2 Department of Nursing, Faculty of Nursing and Midwifery, Kurdistan University of Medical Sciences, Sanandaj, Iran; 3 Department of Public Health, School of Public Health, Qom University of Medical Sciences, Qom, Iran

**Keywords:** Cesarean section, Primigravid women, COVID-19 pandemic, Interrupted Time Series, Kurdistan Province.

## Abstract

**Background:**

Cesarean section (C-section) rates, deemed a critical health indicator, have experienced a historical increase. The advent of the COVID-19 pandemic significantly impacted healthcare patterns including delays or lack of follow-up in treatment and an increased number of patients with acute problems in hospitals. This study aimed to explore whether the observed surge is a genuine consequence of pandemic-related factors.

**Methods:**

This study employs an Interrupted Time Series (ITS) design to analyze monthly C-section rates from March 2018 to January 2023 in Kurdistan province, Iran. Segmented regression modeling is utilized for robust data analysis.

**Results:**

The C-section rate did not show a significant change immediately after the onset of COVID-19. However, the monthly trend increased significantly during the post-pandemic period (*p* < 0.05). Among primigravid women, a significant monthly increase was observed before February 2020 (*p* < 0.05). No significant change was observed in the level or trend of C-section rates among primigravid women after the onset of COVID-19.

**Conclusion:**

This study underscores the significant and enduring impact of the COVID-19 pandemic in further increasing the C-section rates over the long term, the observed variations in C-section rates among primigravid women indicate that the COVID-19 pandemic had no statistically significant impact.

## Background

Cesarean section (C-section) is one of the indicators used to monitor the goals of the Millennium Development Health targets [1] and according to the World Health Organization; the C-section rate should not exceed 15% of total births [2]. In Iran, the Natural Delivery Promotion Package as a part of the health system transformation plan was introduced to improve maternal and neonatal health indicators by reducing the cesarean rates in the country in 2014. Accordingly, it is expected that making natural delivery free in hospitals affiliated with Ministry of Health and Medical Education, obliging hospitals to reduce the C-section rate, will affect the behavior of mothers and health service providers in choosing the mode of childbirth [1, 3]. At the same time, an increase of six-fold in the cesarean rate has been reported from 1970 to 2018. In this way, it increased from 7% to more than 48% in 2018 [4]. It appears that increasing the fee for C-section procedures compared to natural delivery has been a significant factor contributing to the inclination of obstetricians and gynecologists to perform unnecessary C-Sect. [1].

On the other hand, with the spread of the COVID-19 pandemic around the world and the infection of millions of people [5], it seems that the onset of the COVID-19 epidemic has significantly affected the pattern of using services, and cesarean will not be an exception. Widespread fear of exposure to COVID-19, and the implementation of different policies to combat the epidemic led to delays or lack of follow-up in treatment and an increased number of patients with acute problems in hospitals. On the other hand, the emerging pattern of utilizing healthcare services such as for elective surgeries, diagnostic procedures, and other medical interventions, which includes delays or inadequate follow-up in treatment, along with a rise in the number of patients presenting with acute issues in hospitals, has further intensified pre-existing challenges [6]. For example, in Zhang’s study in China at the beginning of the pandemic, the cesarean ratio was significantly higher in areas where confirmed COVID-19 cases were identified or in pregnant women who experienced quarantine [7]. In Margot’s review study; it was found that 68.9% of women infected with COVID-19 gave birth via cesarean. The increase in cesarean rate may reflect obstetricians’ efforts to provide the best care to their patients under COVID-19 conditions [8]. Experts believe that early delivery, even in non-indicated cases, is beneficial for subsequent treatment and outcomes of COVID-19. This has led to an increase in the preterm birth rate (21 − 31%) [9]. Therefore, the impact of COVID-19 as an unexpected event on the health system can be identified by evaluating changes in the level and slope of time series and determining the statistical importance of related parameters.

## Methods

### Research design

The study utilized an interrupted time-series (ITS) design to investigate the rate of C-sections before and during the COVID-19 pandemic in February 2020 in Kurdistan province. ITS is considered the most robust quasi-experimental approach for evaluating the effects of implemented interventions in a before-after scenario [10]. It has been widely employed to assess various public health interventions and the health impacts of unforeseen events [11]. Therefore, in this study, ITS was employed to determine whether there were any differences in the observed C-section rates before and during the COVID-19 pandemic.

### Data collection

We obtained routine hospital monthly data on reproductive services (Natural Delivery& Cesarean) from the HIS system—a local electronic health record. Data were collected in a census sampling from 24 months before the onset of COVID-19 (from March 2018 to February 2020) to 35 months during the COVID-19 pandemic (from February 2020 to January 2023).

### Data analysis

Data were analyzed by a robust interrupted time series (ITS) approach using segmented regression modeling. The interrupted time series method is used for statistical analysis to evaluate the impact of an intervention by observing data before and after the intervention. This approach involves tracking a long-term period, assessing the effects of the intervention, and analyzing any changes in the data over time.

The following specific segmented regression model [12] was used to estimate the effect of the COVID-19 pandemic on the monthly C-section rate [13].

Yt = β0 + β1 ∗timet + β2∗COVID − 19t + β3∗timeafterCOVID − 19t + εt,

In the model, Yt represents the cesarean rate in month t, where t is a time trend variable ranging from 1 to 59 (the first and last observations). The binary variable COVID-19t distinguishes between the pre-pandemic period (COVID-19t = 0) and the post-pandemic period (COVID-19t = 1). For the period after the COVID-19 pandemic, the variable takes values between 1 and 35. In the model, β0 estimates the starting level of the outcome at time zero. β1 estimates the monthly trend in cesarean rate before the pandemic. β2 estimates the immediate change in monthly cesarean rate after COVID-19. β3 estimates the difference in the monthly trend post-COVID-19 compared to pre-existing trend. The Dickey-Fuller test was applied to examine stationarity of time series data, showing no unit root and the stationary series. Version 14.2 of StataCorp software was used for analysis.

## Results

**Fig. 1:**
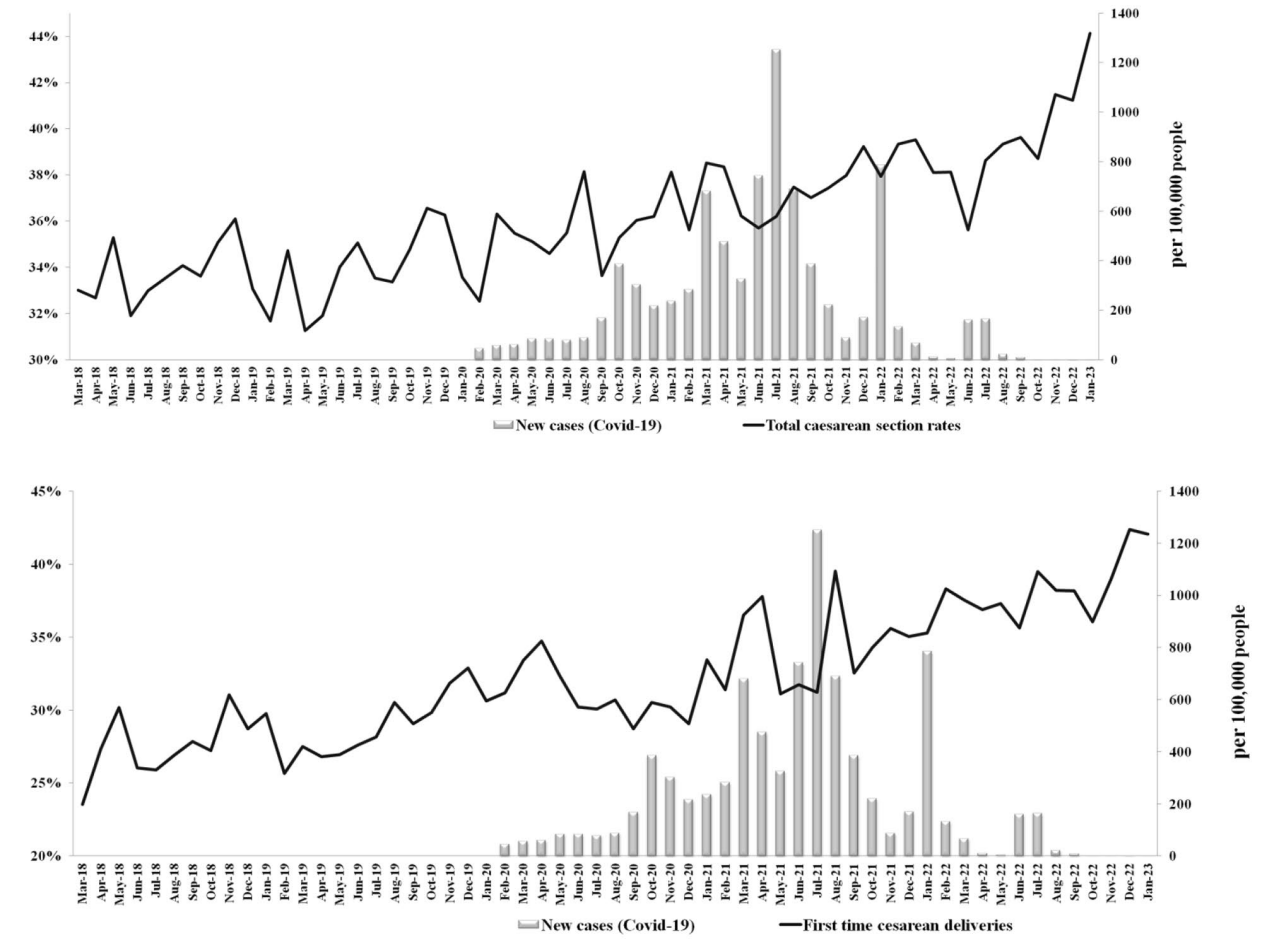
Trend of the monthly C-section rates before and after the onset of COVID-19 (March 2018 to January 2023)

A total of 111,149 deliveries have been performed. Overall, 39,827 cases (35%) were C-section, and 12,793 cases (12% of all deliveries) were reported as the first time cesarean deliveries. The C-section rates before and after the onset of the covid-19 was calculated as 34% and 37%, respectively. Figure 1 illustrates the monthly fluctuations in C-section rates pre- and post-onset of the COVID-19 pandemic from March 2018 to January 2023 among all pregnant women as well as primigravid women. The figure of the total C-section ratio demonstrates a progressive rise after the advent of COVID-19. Likewise, in primigravid women, rates of cesarean delivery initially spiked at the pandemic’s start and generally displayed an upward trend overall.

**Fig. 2:**
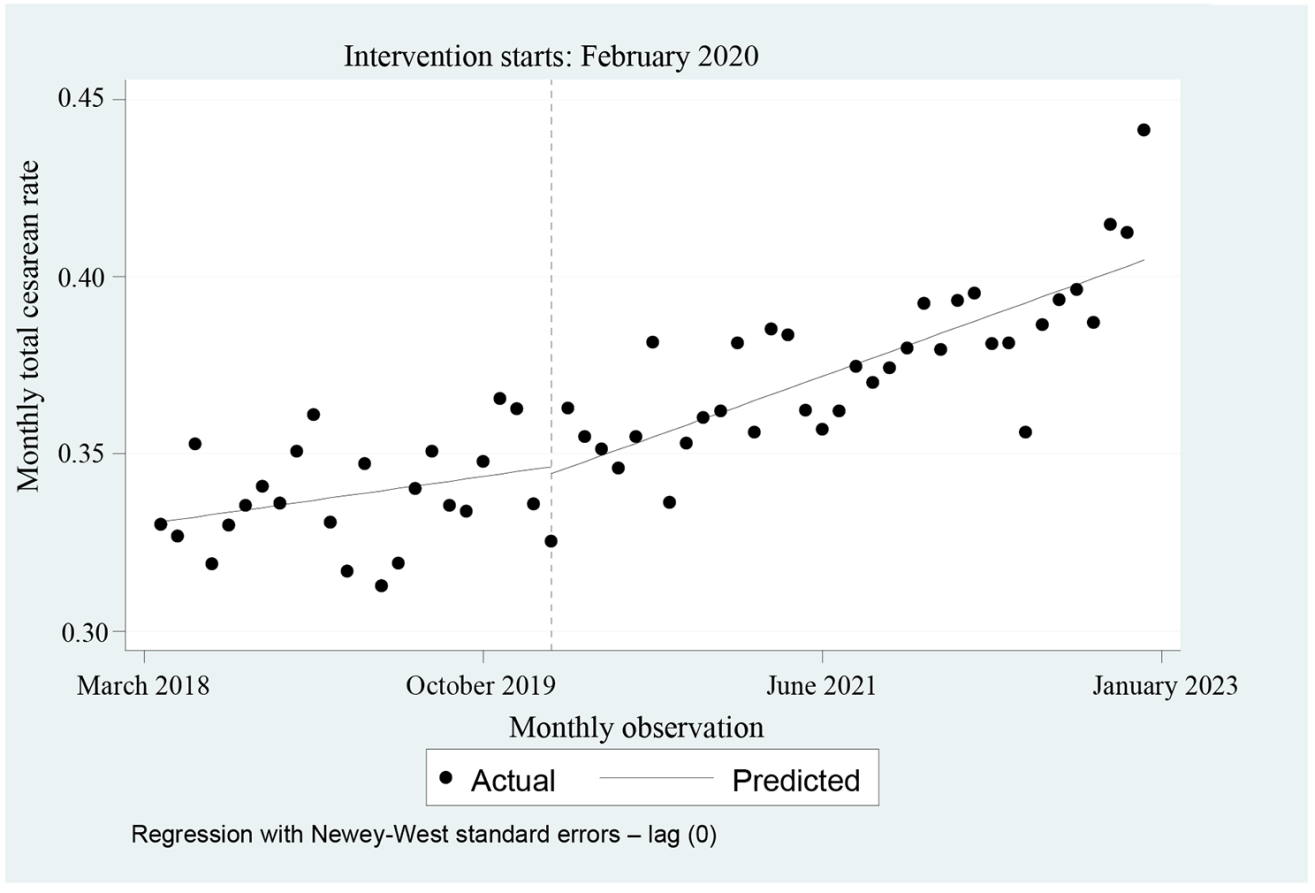
Regression results of the total monthly cesarean rate before and after the onset of COVID-19.

As shown in the regression table (Table 1), the starting level of the cesarean rate was estimated to be 0.330, and the C-section rate appeared to have no significant change every month before February 2020 (*P* = 0.094). Also, right after the intervention (start of COVID-19), we did not observe any significant change in the C-section rate (level or short effect) (*P* > 0.05). But, a significant increase of 0.00105 was observed in the regression slope (Trend or long effect) (*P* = 0.0) (Table 1). Figure 2 presents the visual display of these results.

**Table 1:** Estimated coefficients of segmented regression model for total cesarean rate in the hospitals in Kurdistan province, March 2018–January 2023

Regression with Newey-West standard errors					
Number of observation = 59		F (3, 55) = 41.97		Prob > F = 0.0000	
Parameter	Coefficient	Newey-West Std. Err.	t	*P*-value	95% confidence interval
Intercept	0.33020	0.00510	64.68	**0.000**	0.3199, 0.3404
Pre-intervention slope	0.00067	0.00039	1.70	0.094	-0.0001, 0.0014
Change in intercept	-0.00197	0.00779	-0.25	0.802	-0.0175, 0.0136
Change in slope	0.00105	0.00048	2.17	**0.034**	0.0000, 0.0020
Post-intervention Linear Trend					

**Fig. 3:**
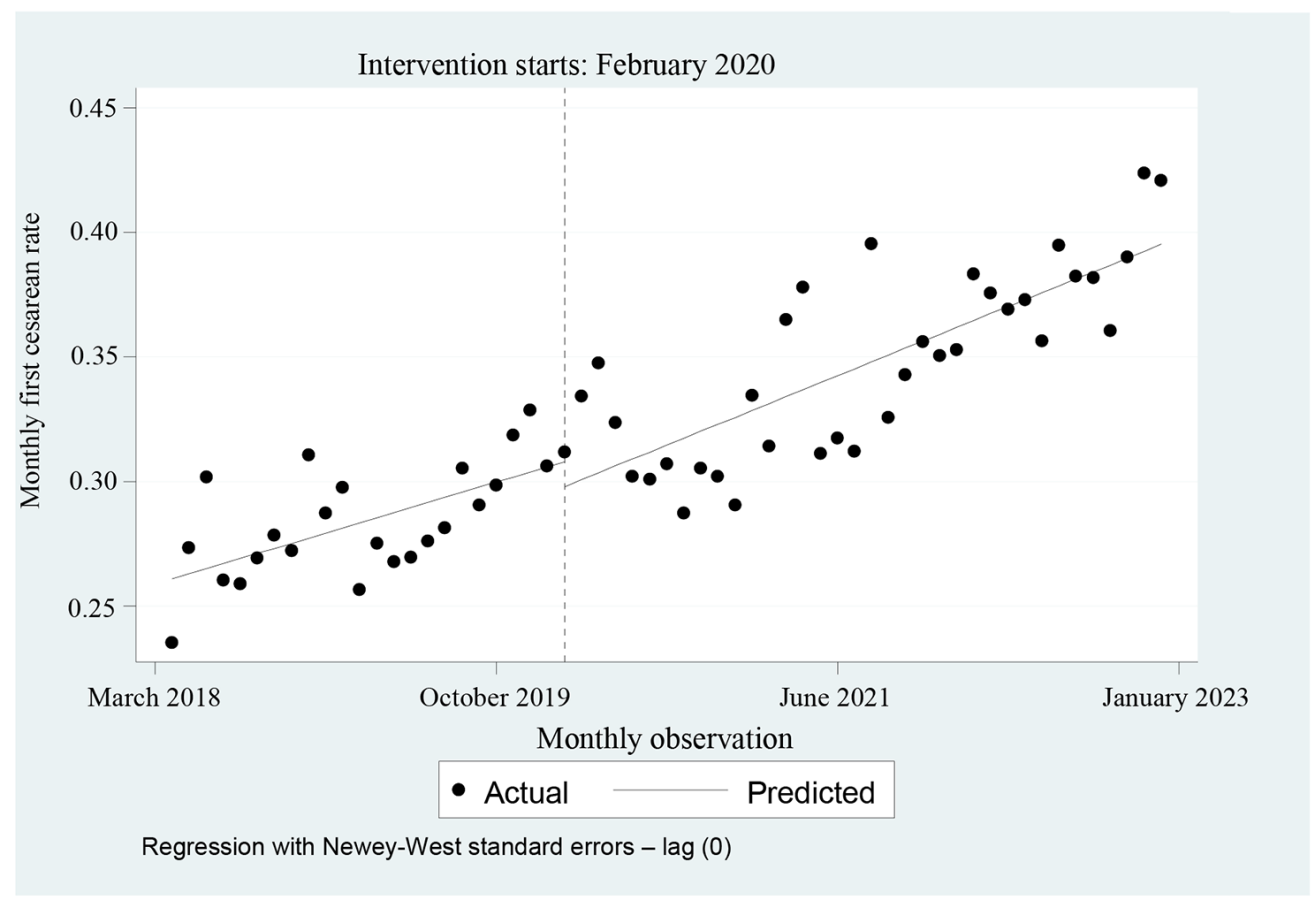
Regression results of the monthly cesarean rate before and after the onset of COVID-19 in primigravid women

As shown in the regression Table 2, the starting level of the C-section rate among primigravid women was estimated to be 0.258, and the C-section rate appeared to significantly increase by 0.002 every month before February 2020 (*P* = 0.001). However, we did not observe any significant change in the level (Short effect) and slope (Long effect) of the regression model after the start of COVID-19 (*P* > 0.05). Figure 3 presents the visual display of these results.

**Table 2:** Estimated coefficients of segmented regression model for cesarean rate among primigravid women in the hospitals in Kurdistan province, March 2018–January 2023

Regression with Newey-West standard errors					
Number of observation = 59		F (3, 55) = 78.51		Prob > F = 0.0000	
Parameter	Coefficient	Newey-West Std. Err.	t	*P*-value	95% confidence interval
Intercept	0.25890	0.00870	29.75	**0.000**	0.2414, 0.2763
Pre-intervention slope	0.00204	0.00058	3.52	**0.001**	0.0008, 0.0032
Change in intercept	-0.00998	0.10905	-0.92	0.364	-0.0318, 0.0118
Change in slope	0.00073	0.00068	1.06	0.293	0.0006, 0.0021
Post-intervention Linear Trend					

## Discussion

Present studies indicate a significant influence of the COVID-19 pandemic on a sustained surge in C-section rates. Prior investigations within Iran have similarly unveiled a substantial upswing in C-section prevalence amid the widespread prevalence of COVID-19 [14]. In the United Kingdom, an aggregate augmentation in the overall incidence of C-section deliveries has been documented during the pandemic [15]. Li, in a report, highlighted an escalation in C-section procedures prompted by maternal requests amid the COVID-19 outbreak [16]. Noteworthy is the case of Turkey, where the C-section rate has experienced an increase from 57.7% in 2019, the pre-pandemic epoch, to 60.2% in 2020 [17]. On the contrary, in New York, there was a noticeable decrease in natural and vaginal deliveries, which can be attributed to the cautious measures implemented by healthcare professionals to reduce the risk of viral transmission. It appears that there was a perception that opting for C-section procedures could potentially lower the likelihood of disease transmission [18]. The discerned escalation in C-section rates may be indicative of apprehensions related to parturition settings and the fear of contracting COVID-19 among pregnant women, coupled with the potential contagion risk within maternity facilities [19, 20]. To elucidate, the expeditious nature of C-sections serves to diminish the parturient’s waiting duration, thereby mitigating the time of exposure to hospital environments during the ongoing pandemic [21]. Furthermore, diverse strategies implemented to combat the COVID-19 epidemic may potentially lead to treatment delays or non-adherence, consequently contributing to a surge in acute cases within hospital settings and, consequently, an elevated propensity for C-section deliveries [9]. Antecedent investigations have also underscored that restricted access to maternity care facilities amplifies the likelihood of C-section procedures [7, 22]. Moreover, insufficient accessibility to natural childbirth services for women with prior C-section histories may act as a catalyst for the observed surge in C-section rates during COVID-19. This escalation might also be emblematic of obstetricians’ concerted endeavors to deliver optimal care under the challenging circumstances posed by COVID-19 [23]. Nevertheless, empirical evidence suggests that pregnant women afflicted with COVID-19 may be predisposed to elevated rates of perinatal and maternal complications, encompassing preterm labor, preeclampsia, and the necessity for C-section deliveries [24].

In the context of primigravid women, the present study findings reveal an ascending trajectory in C-section rates. However, the influence of COVID-19 on the C-section rate within this cohort appears to be inconsequential. These findings bear enhanced credibility, given that, within the broader spectrum of delivery outcomes, the rationale for the escalating C-section rates may be traced back to the enduring impact of antecedent C-sections. Previous research has underscored that primary C-sections frequently precipitate subsequent secondary C-sections, with attempts at vaginal delivery after a C-section being concomitant with an augmented risk of emergent C-section, uterine rupture, and failed vaginal delivery [25–27]. The trend of C-section rates among primiparous women in China signifies a noteworthy surge, escalating from 19.1% in 1995 to 43.2% in 2019 over the past quarter-century, attributed to the one-child and two-child policies [28]. In light of the present study, it is evident that COVID-19 had minimal impact on the C-section rates among primigravid women. Therefore, the C-section rates among first-time pregnant women should be considered as a more significant and accurate metric for assessing the influence of COVID-19 on C-section deliveries. Consequently, it can be inferred that the changes in C-section rates were primarily driven by the history of prior C-sections rather than the effects of COVID-19. These discernments assume paramount importance, as they intimate that the repercussions of COVID-19 on C-section rates may be entwined with factors beyond the inaugural pregnancy experience. Notwithstanding the implementation of Health Transformation Plan (HTP) policies in recent years, designed to curtail C-section rates and advocate for vaginal deliveries, with empirical studies documenting a diminishing trend post-HTP initiation [29], the envisioned annual reduction of 10% remains unrealized [30]. Even after the execution of HTP initiatives, a surge in C-section rates has been witnessed within certain private healthcare facilities, corroborated by the present inquiry. Analogous occurrences have been recorded in other global locales. For instance, in a Peruvian study, post-health sector overhauls and alterations in remuneration mechanisms, the C-section rate burgeoned from 28 to 53% [31]. In Uruguay, heightened remunerations to private practitioners rendered the C-section rate more than double that of their public sector counterparts [32].

Despite the considerable amount of available data, this study has the certain limitations, including the unavailability of crucial variables about women’s sociodemographic characteristics, such as age, education, socioeconomic status, and ethnicity. These variables, known to influence cesarean section rates, were not included in the analysis, potentially impacting the study’s findings. These variables have been previously established as influential factors in cesarean section rates. Additionally, the study does not account for the prevalence of COVID-19 among pregnant women before and during pregnancy, which may have contributed to variations in cesarean section rates. Understanding the impact of COVID-19 infection on pregnancy outcomes and cesarean section rates is essential for comprehensive analysis.

## Conclusions

The findings of this study indicate that although the rate of cesarean sections was consistently on the rise, there was no significant association between COVID-19 and its impact on primiparous women. The observed variations in C-section rates among this specific population suggest that the COVID-19 pandemic did not have a statistically significant effect on them. Consequently, considering the potential influence of COVID-19 on the overall cesarean rate, in conjunction with other factors such as prior cesarean deliveries and the inadequacy of past interventions to decrease the cesarean rate in Iran, it is imperative to conduct precise, comprehensive analyses and further research to comprehend the intricate factors contributing to the escalating cesarean rate and its enduring repercussions. Moreover, additional research is required to gain a better understanding of the long-term consequences of this heightened rate and to emphasize the implementation of more effective measures to reduce unnecessary cesareans. Although this study was conducted in the Kurdistan province of Iran, its outcomes may impact policymakers and healthcare providers not only in Iran but also in analogous regions, urging them to reassess and formulate effective strategies to diminish the prevalence of C-section.

## Data Availability

The datasets used and/or analyzed during the current study are available from the corresponding author on reasonable request.
